# Clonal Human Fetal Ventral Mesencephalic Dopaminergic Neuron Precursors for Cell Therapy Research

**DOI:** 10.1371/journal.pone.0052714

**Published:** 2012-12-31

**Authors:** Tania Ramos-Moreno, Javier G. Lendínez, María José Pino-Barrio, Araceli del Arco, Alberto Martínez-Serrano

**Affiliations:** 1 Department of Molecular Biology (U.A.M.) and Center of Molecular Biology “Severo Ochoa” (U.A.M.-C.S.I.C.). Nicolás Cabrera, 1 Universidad Autónoma de Madrid, Campus Cantoblanco, Madrid. Spain; 2 Área de Bioquímica, Centro Regional de Investigaciones Biomédicas (CRIB), Facultad de Ciencias Ambientales y Bioquímica, Universidad de Castilla-La Mancha, Toledo, Spain; University of Freiburg, Germany

## Abstract

A major challenge for further development of drug screening procedures, cell replacement therapies and developmental studies is the identification of expandable human stem cells able to generate the cell types needed. We have previously reported the generation of an immortalized polyclonal neural stem cell (NSC) line derived from the human fetal ventral mesencephalon (hVM1). This line has been biochemically, genetically, immunocytochemically and electrophysiologically characterized to document its usefulness as a model system for the generation of A9 dopaminergic neurons (DAn). Long-term *in vivo* transplantation studies in parkinsonian rats showed that the grafts do not mature evenly. We reasoned that diverse clones in the hVM1 line might have different abilities to differentiate. In the present study, we have analyzed 9 hVM1 clones selected on the basis of their TH generation potential and, based on the number of *v-myc* copies, *v-myc* down-regulation after *in vitro* differentiation, *in vivo* cell cycle exit, TH^+^ neuron generation and expression of a neuronal mature marker (hNSE), we selected two clones for further *in vivo* PD cell replacement studies. The conclusion is that homogeneity and clonality of characterized NSCs allow transplantation of cells with controlled properties, which should help in the design of long-term *in vivo* experiments.

## Introduction

The development of neural transplantation as a treatment for Parkinson’s disease moved to the clinics after obtaining convincing results in animal models of Parkinson’s Disease (PD). Clinical research on neuron replacement using human fresh ventral mesencephalon tissue (hfVM) provided proof of principle of the therapeutic efficacy of dopaminergic transplants on a long-term basis [1]. Nevertheless, the limited supply of hfVM, together with the variability observed in the outcome of different clinical trials, along with the appearance of graft-induced dyskinesias (GIDs), lead to the current need of developing strategies to reproducibly generate large numbers of neural cells in a safe manner, and under standardized and controlled conditions (recently reviewed in [1], [2], [3], [4], [5], [6], [7]. Stem cells promise to be such source of cells. Importantly, maturation of human neurons and of DA neurons (DAn) from their precursors is a lengthy process, both *in vitro* and *in vivo*, in experimental animals and humans, requiring at least 5–6 months to take place [8], [9], [10], [11], [12]. Therefore, experiments to document the possible *in vivo* therapeutic efficacy of stem cells and their derivatives, along with safety, need to be carefully addressed in experimental animals on a long-term basis [13], [14], [15].

At present, the experimental confirmation that transplants of human Neural Stem Cells (hNSC) of VM origin can lead to long-term (6–12 months) striatal reinnervation, substantial DA replacement and behavioral recovery with a high safety profile, is still missing [1], [2], [16].

Our group has recently generated and characterized an immortalized model cell line of VM hNSC called hVM1, a reliable source of human A9-subtype DAn, both *in vitro* and *in vivo* [17], [18]. This non-transformed cell line generates a substantial percentage of DAn after *in vitro* differentiation (about 12% of total cells), which show typical properties of Substantia Nigra *pars compacta* (A9-subtype) DAn (reviewed in ref. [18]; see also refs. [8], [19]. Behavioral efficacy of transplants of these VM hNSCs has been consistently reported in the unilateral complete 6-OH-DA rat PD model. Thus, the transplants result in amelioration of not only drug (amphetamine, apomorphine)-induced rotational asymmetry but also spontaneous behavior (e.g., dexterity in the staircase or paw reaching test) [19], [20]. These VM hNSCs loose their neurogenic capacity with passaging [20], as reported by other groups for both rodent and human VM DAn precursors [21], [22], [23], [24], [25], [26], [27]. We solved this drawback by augmenting cellular Bcl-X_L_ levels [8], [19], [20]; reviewed in [18].

The present work aims at the identification of a homogeneous, clonal source of VM hNSCs and resultant human DAn with optimal properties for drug screening, developmental studies and, most of all, cell therapy research. The study was motivated by the fact that functional maturation of human DAn *in vivo* requires long survival times, as explained above, and, when studying the histology and anatomy of transplants of hVM1 cells, graft maturation was found to be uneven [19]. In addition, major differences have been observed between transplants of these midbrain hNSCs and previous transplantation studies using forebrain hNSCs. Whereas forebrain hNSCs smoothly integrate and migrate into the brain parenchyma from the implantation site (both neurospheres and immortalized cells, refs. [12], [28], [29], both hfVM and VM hNSCs show more limited migration, generating compact transplants [10], [19], [20], [30], [31], [32]. In the case of hVM1 cells, we have observed a rather immature morphology of the DAn at 2 months post-grafting, and also areas of the transplants that were evidently not maturing even at four months post transplantation (see for instance neuronal morphologies in Fig. 9 in ref. [20] and Fig. 3 in ref. [19]. In addition to the lengthy differentiation process of human DAn, we hypothesized that difficulties to achieve full maturation could arise if some of the sub-clones composing the hVM1 cell line had different maturation rates, or continued in proliferation, even if that would occur at a slow rate. To clarify this and to provide homogenous material for future studies, we have characterized in detail 9 out of 70 previously isolated sub-clones of the hVM1 polyclonal line [17], previously chosen on the basis of their TH^+^ neuron generation rate. Thus, we have identified the best clone candidates for *in vivo* PD cell replacement preclinical research based on the number of *v-myc* copies in the genome, *v-myc* down-regulation after *in vitro* differentiation, cell cycle exit 2 months after grafting, generation of TH^+^ cells both *in vitro* and *in vivo* together with the expression of a mature neuronal marker (human neuron specific enolase, hNSE). The selected clones show marked cessation of cell division in comparison with the polyclonal cell line and induce partial amelioration of behavioral asymmetry even at the short time point studied here (2 months). We expect that these clonal and homogeneous VM hNSCs will allow carrying out long-term transplantation studies to unambiguously determine the safety and efficacy of VM hNSCs for cell replacement in experimental PD.

## Materials and Methods

### Ethics Statement

Principles of both laboratory animal care and use of stem cells were approved by the relevant Research Ethics Comitees, these being the following: Autonomous University Comitee for Ethical Research (ref. CEI 23–564), Comision de Seguimiento y Control de la Donacion y Utilizacion de Celulas y Tejidos Humanos (ref. 123-101-1), Comite Etico de Investigacion Clinica Regional de la Comunidad de Madrid (ref. OPTISTEM-DA).

All animal work has been designed following the 3Rs principles. Details of animal work and welfare are described under the Animal Experimentation section below.

Ethics statements about the human fetal origin of the cells used in the present study can be found in the original reports describing hVM1 and hNS1 cell lines [17], [33].

### Cell Culture Procedures

VM hNSCs (polyclonal line hVM1) overexpressing and non-overexpressing Bcl-X_L_ and 9 different hVM1 clones pre-isolated in terms of their TH generation *in vitro* were routinely cultured on 10 µg/ml poly-L-lysine -pretreated plastic-ware as previously described [17], [20]. Cells were grown at 37°C, in a 95% humidity, 5%CO2 and 5% O2 atmosphere (low oxygen conditions). For inmunocytochemistry assays, the cells were grown in 50 µg/ml poly-L-lysine treated sterile round glass cover-slips (0.1 mm thickness, 13 mm diameter). Differentiation experiments were performed as described elsewhere [20]. As a control in some *in vitro* experiments we used forebrain-derived, immortalized clonal hNSCs (cell line hNS1, formerly HNSC.100), cultured and differentiated as described [33].

### Genomic DNA Isolation and Southern Blot

Genomic DNA was isolated by incubating cell pellets in lysis buffer (2% Tris-HCl 1M pH 8, 2% NaCl 5M, 5% SDS 10%, 2% EDTA 0.5M) containing proteinase K (20 mg/ml) overnight at 55°C. After that, samples were mixed with 300 µl of saturated NaCl solution and incubated 10 min at 4°C, followed by centrifugation at maximum speed 20 min. The supernatant was mixed with 800 µl of iso-propanol and incubated on ice 15 min. Samples were centrifuged again at maximum speed 20 min and the pellet rinsed with 70% ethanol, dried and dissolved in water. For Southern Blotting 10 µg of DNA were digested with EcoRI (4 h, 37°C). After electrophoresis, DNA was transferred to a nylon membrane (Hybond N; Amersham) and hybridized with a 1.3 Kb SacI-BamHI [α-^32^P] dCTP labeled probe [33].

### Protein Extraction and Western Blot (WB) Assays

Total protein was extracted from cultures under proliferation and also after 10 days of differentiation. Blots were incubated overnight at 4°C with the following primary antibodies: *v-myc* (rabbit-polyclonal, Upstate Biotechnology, 1∶400), β-actin (mouse-monoclonal, Sigma, 1∶10000). Immunoreactivity was detected using the ECL Western blot detection system (Amersham Biosciences) and the blots were quantified by scanning densitometry using ImageJ (NCBI/NIH) software.

### Immunocytochemistry (ICC)

Cultures were fixed under proliferation and after 10 days differentiation conditions with 4% buffered paraformaldehyde. Samples were incubated overnight at 4°C in PBS containing 1% goat serum, 0.25% Triton X-100 with the following primary antibodies: Ki-67 (Thermo Scientific, 1∶200), Nestin (BD Transduction Laboratories, 1∶600), β-III-tubulin (Sigma, 1∶2000) or TH (Chemicon, 1∶1000). Cultures were rinsed and incubated for 1 h with fluorescent secondary antibodies: Cy3-conjugated goat anti-mouse for nestin and β-III-tubulin (1∶200, Jackson Inmunoresearch) and Cy5-conjugated donkey anti-rabbit for Ki-67 and TH (1∶200, Jackson Inmunoresearch) and Hoechst 33258 (1∶400, Molecular Probes) to label nuclei. Samples were coverslipped with Mowiol. Microscopic examination and photography of the samples were performed by epifluorescence microscopy, and analyzed with ImageJ (NCBI/NIH) software.

### Animal Experimentation: Lesion, Drug-induced Rotation and Transplantation Procedures

Experiments were carried out according to the guidelines of the European Community (Directive 86/609/ECC) and in accordance with the Society for Neuroscience recommendations. Animals used in this study were 3-month-old female Sprague-Dawley rats (Harlan), weighing 200–250 g at the beginning of the experiment.

We chose groups of reduced number of rats (3–5) to apply the *Reduction* concept of the 3 Rs principle [34] in view of the large number of clones to study. Although this low number of animals is insufficient to generate behavioral data with statistical power, the most important part of the *in vivo* studies is the histological analysis, and for this purpose a number of 3–5 rats suffices.

Animals were housed in a temperature- and humidity-controlled room, under 12 hours light/dark cycles, with *ad libitum* access to food and water. Rats were anesthetized with a ketamine-xylacine cocktail for all lesion and transplantation surgeries. Rats received a 6-hydroxydopamine (6-OH-DA) injection (9 µg/3 µl dissolved in 0.9% saline containing 0.2 mg/ml ascorbic acid; Sigma) in the right median forebrain bundle at the following stereotaxic coordinates (tooth bar set at −3.3 mm): antero-posterior, −3.7 mm; medio-lateral, −1.6 mm (both from bregma); dorso-ventral, −8.8 mm from dura. The injection rate was 1 µl/min, and the syringe was kept in place for an additional 5 min before being slowly retracted. Four weeks after the lesion, the rats were tested for rotational behavior in automated rotometer bowls (Panlab) following an intraperitoneal injection of D-amphetamine sulfate (5 mg/kg, Sigma). Rotation scores were collected every 2 min for 90 min in a computer-assisted rotometer system (Panlab). Only rats exhibiting 5 or more ipsilateral rotations/min after D-amphetamine injection were selected for further transplantation studies. Balanced groups of 3–5 hemiparkinsonian rats were transplanted with the 9 different clonal hVM1 cell lines and hVM1 polyclonal cell lines (over-expressing or non-overexpressing Bcl-X_L_; 17, 20) in proliferative state. Cells were trypsinized and resuspended in Ca^2+^ and Mg^2+^ containing Hanks’ balanced salt solution (Invitrogen) at a density of 10^5^ cells/µl. Three µl of cell suspensions (a total of 3×10^5^ cells) were injected into the denervated striatum at the following coordinates (in mm): antero-posterior, +1; medio-lateral, −3; dorso-ventral, −4.5 (from dura), with the tooth bar set at −2.3. The animals were immunosuppressed with daily intraperitoneal cyclosporine A injection (15 mg/kg; Novartis), starting 2 days before transplantation and throughout the experiment. Seven weeks after transplantation, the animals were anesthetized with an overdose of chloral hydrate and intracardially perfused with freshly prepared, buffered 4% paraformaldehyde (in 0.1M phosphate buffer, pH 7.4).

### Immunohistochemistry (IHC)

Brains were removed after animal perfusion, post-fixed for 12 h in the same fixative at 4°C, and dehydrated in 30% sucrose solution at 4°C until sunk. Ten series of 30 µm-thick coronal sections were collected using a freezing microtome. Serial sections were used for immunohistochemistry with polyclonal antibodies against TH (1∶400; Pel-freeze) and monoclonal antibodies against human nuclei (HuNu) (1∶100; Chemicon), Ki-67 (1∶200, Thermo Scientific), human neuron-specific enolase (hNSE) (1∶1000; Chemicon). Secondary abtibodies used were biotinylated goat anti-rabbit (1∶200, Vector), biotinylated rabbit anti-sheep (1∶200, Vector), donkey anti-mouse CY5 (1∶200, Jackson Inmunoresearch) and goat anti-rabbit CY3 (1∶200, Jackson Inmunoresearch), depending on the primary antibody. The sections incubated with biotinylated secondary antibodies were then incubated with a complex of avidin-biotin-peroxidase (ABC kit, Vector Laboratories) and developed with 1,3-diaminobenzidine (DAB, Sigma). Finally, the sections were mounted onto gelatinized glass slides (Menzel-Glaser), dried overnight and coverslipped with Mowiol for the fluorescent IHC or 1,3-diethyl-8-phenylxanthine (DPX) for the DAB IHC.

### Imaging and Data Analyses

Analysis and photography of fluorescent or DAB stained samples were carried out in an inverted Zeiss Axiovert 135 (Oberkochen, Germany) or Leica DM IRB microscope equipped with a digital camera Leica DC100 (Nussloch, Germany). In some of the immunofluorescence experiments, digitized images were captured using Leica IM500 or LSM710 software. Image analyses were performed using NIH Image J software.

### Quantification of HuNu^+^ and Ki-67^+^ Cells in the Grafts

In order to assess the extent of cell cycle exit *in vivo*, we quantified the co-localization of HuNu (Human Nuclei) and Ki-67 protein (cellular marker for proliferation) using NIH Image J software. Briefly, the whole extent (medio-lateral and dorso-ventral) of the grafted striatum was analyzed by confocal microscopy in sections spaced 240 µm (antero-posterior).

### Statistics

*Student t-tests* were ran out to compare data between groups using Statistika software. A significance level of p<0.001 and p<0.05 was chosen for *Student t-test* one tail.

## Results and Discussion

**Figure 1:**
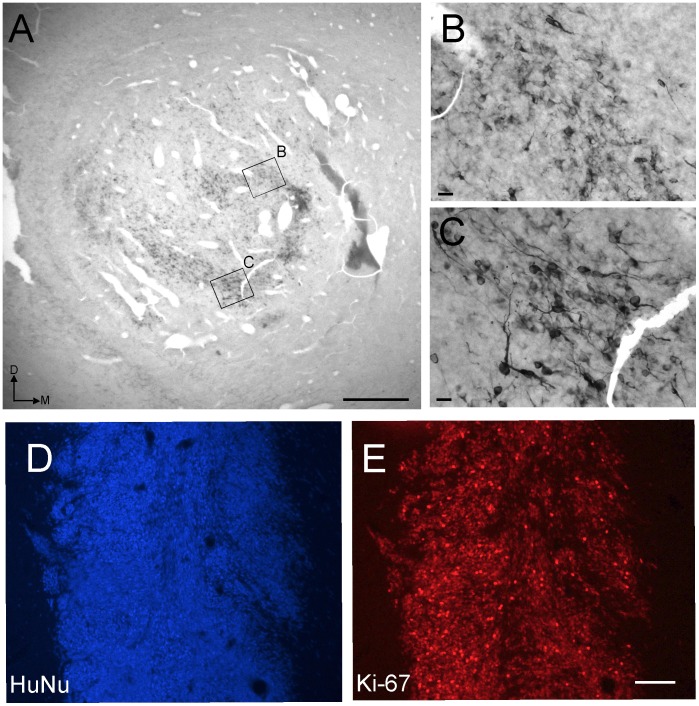
*In vivo* maturation of VM hNSC transplants. VM hNSCs (hVM1-Bcl-X_L_) were transplanted into the 6-OH-DA lesioned rat striatum for a survival time of 2 months. **A–C)** Tyrosine hydroxylase immunohistochemistry developed with DAB, showing areas in the graft populated with morphologically immature TH^+^ neurons (**B**) and others with mature neurons (**C**), or devoid of them. **D**, **E**) Numerous cells in the grafts were still engaged in the cell cycle (Ki-67^+^, panel **E**) at this survival time. Scale bar in A, E: 500 µm; in B, C: 20 µm.

As pointed out in the Introduction, analysis of polyclonal hVM1 or hVM1- Bcl-X_L_ cell transplants at both 2 and 4 months post-grafting revealed an immature morphology of TH^+^ neurons, together with areas of the transplants devoid of TH^+^ neurons, a finding that was confirmed in the present study (see Figure 1A–C). We processed the transplanted brains (2 months post-grafting) for HuNu and Ki-67 antigens, finding that there were areas in the transplants in which the cells were actively engaged in cell division (Ki-67^+^) (see Figure 1 D, E). Furthermore, in the worst cases, the grafts did appear as large, rounded masses of tissue resembling to what could be defined as overgrowths. Cell karyotype was however normal (46XY, no structural abnormalities) in the cells, even at later passages than those used here for transplantation (pass 30, Ramos-Moreno, *unpublished*).

Because of these reasons and in order to identify optimal VM NSCs for future research, we decided to screen a library of clones that were isolated at the time of generating the parental and polyclonal cell line hVM1 [17]. Thus, out of a total of 70 clones, we have chosen 9 clones on the basis of a good rate of TH^+^ generation to be compared on a number of criteria to the polyclonal line. Since the hVM1 line overexpressing Bcl-X_L_ shows a high rate of DAn generation (up to 15–20% of the total cells, see refs. [8], [20], we have considered this line as a reference cell line. Thus, the hVM1- Bcl-X_L_ cells become the “gold-standard” for the screening process.

### V-myc Copy Numbers and Down-regulation of v-myc Expression

A poor *in vivo* down regulation of *v-myc* expression in the cells (the *v-myc* coding retroviral vector gets silenced when the cells are not exposed to mitogens, see refs. [17], [33], [35], [36] could be one of the reasons explaining retarded differentiation and/or persistent cell division of the polyclonal hVM1 and hVM1-Bcl-X_L_ cells. If multiple copies of the vector co-exist in the same cells, the likelihood of having too high expression level of *v-myc* increases, either because of accumulation of the residual mRNA from multiple copies of the vector, or because the probability of having one or more copies not well down-regulated increases.

**Figure 2:**
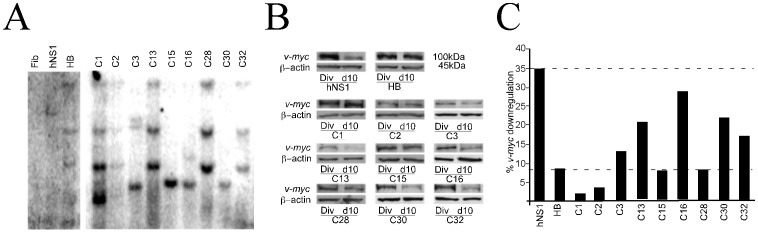
*v-myc* integrations and downregulation upon differentiation. A) Southern blot showing the number of integrated copies of v-*myc* vector in each clone and cellular lines used as controls. Human fibroblasts (Fib), negative control; clonal human forebrain neural stem cells (hNS1), known to have only one insertion [35]; polyclonal hVM1-BclX_L_ cell line (HB) expected to show multiple integrations since it is composed of a library of clones. **B** ***)*** Examples of Western blots showing v-*myc* protein detection. Samples of total protein were extracted under proliferation (Div) and after 10 days of differentiation (d10) conditions for each clone and control cell lines. Note that the clonal hNS1 cells downregulate vector expression whereas the level of v-myc protein is still high in the HB cells after 10d of differentiation. **C)** *v-myc* protein signal in the blots was quantified using Image J software. The degree of downregulation (10 d/Div) is represented. The extent of down-regulation of v-*myc* protein obtained for hVM1-Bcl-X_L_ cells (HB) was 8.3% and was taken as the minimum acceptable down-regulation that a clone should have. Thus, clones with a downregulation over 8.3% were considered. The value obtained for the downregulation of the clonal forebrain hNS1 cell line, 34.7%, would be the ideal or maximal downregulation.

The number of v-*myc* retroviral integrations in the genome were thus studied in the hVM1 sub-clones. Ideally, the most suitable clone should contain a single v-*myc* integration at a chromatin region favoring proper down-regulation of expression upon differentiation (as previously reported for the clonal hNS1 cells, refs. [33], [35]. Such a clone would be preferred in comparison to clones bearing more than one v-*myc* copy. We have used human fibroblasts as a negative control; the polyclonal hVM1- Bcl-X_L_ cell line as a cell line likely having multiple v-*myc* integrations, and the clonal human neural stem cell line (hNS1) known to have only one v-*myc* insertion [33]. In the case of the polyclonal hVM1- Bcl-X_L_ cells, as expected, the southern blot indicates multiple copies of *v-myc* (Figure 2A). Regarding clones, nearly all of them have more than one insertion, but for clone numbers 15 and 30 (Figure 2A and Table 1). Some of the clones such as number 13, 28 and 32 seem to be sister clones.

**Table 1:** Global account of the results obtained for the different criteria used.

	Polyclonal	Clone 1	Clone 2	Clone 3	Clone 13	Clone 15	Clone 16	Clone 28	Clone 30	Clone 32
v-*myc* copies	4	4	2	3	2	1	2	2	1	2
v-*myc* downregulation	+	−	−	+	++	−	++	−	+	+
*Ki*-67 downregulation	++	+	+	−	−	−	−	−	+	++
*Nestin* downregulation	+	−	+	++	+	−	−	−	−	+
Differentiation to *ß-III-tub^+^* cells	+	+	++	+	++	+	−	−	+	++
Differentiation to *TH^+^* cells	+	+	++	-	++	+	−	−	+	++
Reduction of *Ki*-67 in vivo	1	1	1.2	1	2.3	3	1.8	1.5	3	3
*hNSE* in vivo	+	nd	nd	nd	nd	nd	nd	nd	+	+
*TH* in vivo	++	+	+	+	+	+	+	−	+	+
Rotational compensation	+	++	+	+	+	+	+	+	++	+

Polyclonal = hVM1-Bcl-XL cells. Scoring is presented from low values (−), to one or two (+) signs, being (++) the best result for each parameter. For the in vivo reduction of Ki- 67 staining, grafts were scored for 1 to 3, being 3 the best score indicating cell cycle exit.

Since the extent of silencing of different *v-myc* integrated copies cannot be predicted, we determined the actual content in *v-myc* protein before and after differentiation. Total protein samples from the polyclonal hVM1-Bcl-X_L_, the monoclonal hNS1 line and the hVM1 sub-clones were extracted from proliferating cells and 10 days following differentiation, and assayed for *v-myc* protein content (Figure 2B). The extent of v-*myc* down-regulation obtained for each clone was compared to that shown by hVM1-Bcl-X_L_ and hNS1 cells. The down-regulation in the polyclonal cell line is considered as the minimum acceptable (8.3%), and that in hNS1 cells as the ideal one (refs. [33], [35]; Figure 2C). Therefore, clones down-regulating in the accepted interval were # 3, 13, 16, 30, and 32 (Figure 2C and Table 1). As noted before, the number of insertions not always correlates with the downregulation of the protein.

### Ki-67 Expression Under Differentiation Conditions

**Figure 3:**
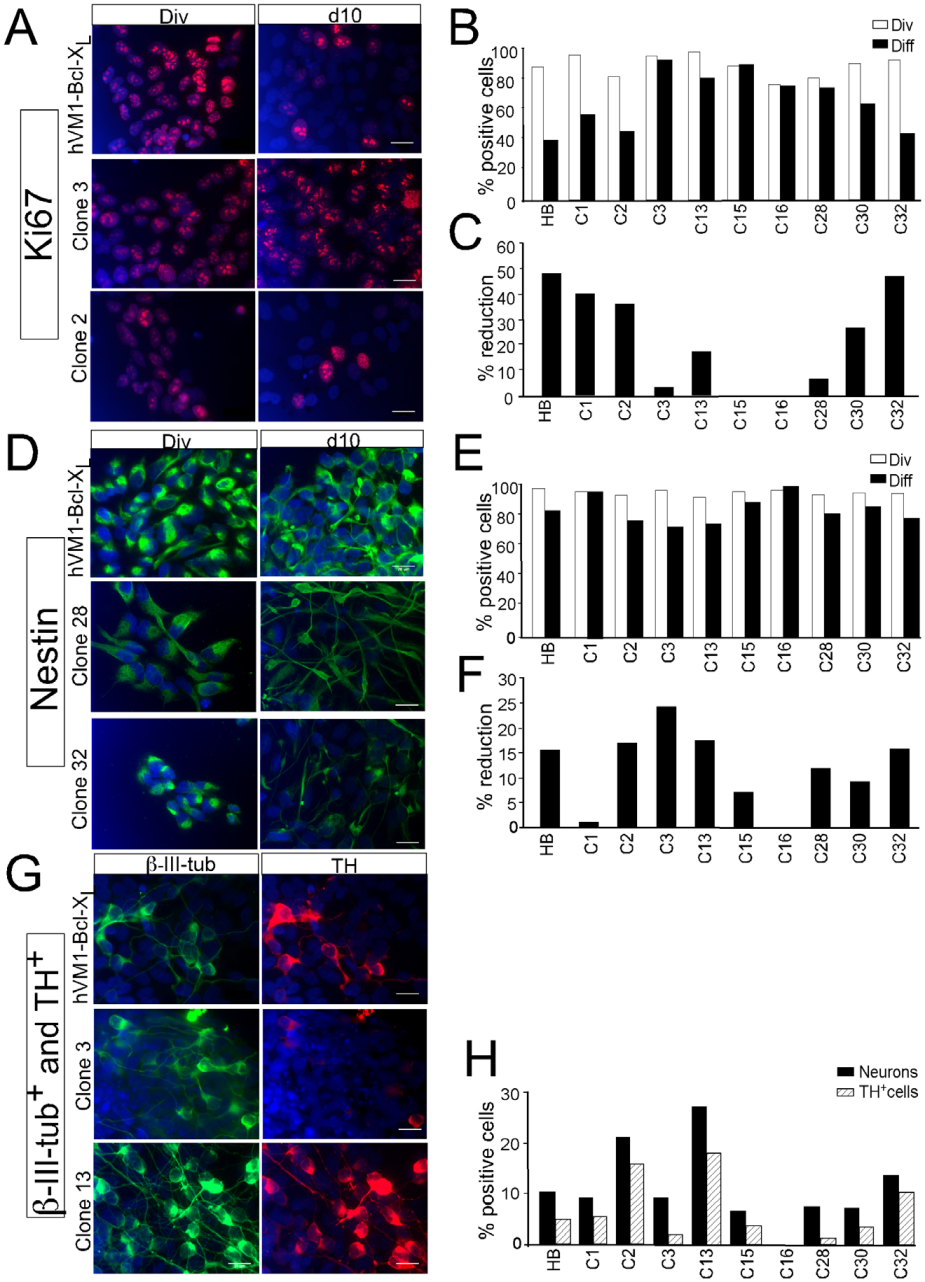
Analysis of Ki-67 and Nestin expression downregulation, and appearance of neuronal markers following differentiation. Ki-67 (**A**) and Nestin (**D**) immunofluorescence of the control hVM1-Bcl-X_L_ cells (HB) and representative clones under division (Div) and following differentiation (d10). In both cases, examples are shown of a clone not showing proper downregulation (#3, 28), and of clones showing good cell cycle exit and disappearance of the NSC marker Nestin (#2, 32). **B** and **E**) Quantitative raw data are shown for control HB cells and all clones. **C** and **F**) Percentage of reduction in the number of positive cells for each marker. **G**) Immunofluorescence for β-III-tubulin and TH of the control hVM1-Bcl-X_L_ cells (HB) and representative clones after differentiation (d10). Note the difference in neurogenic and DAn generation capacity for the different clones (**H**). Clones # 32, 2 and 13 generate more TH^+^ cells than hVM1-Bcl-X_L_ cells. Clones # 1, 15 and 30 are close to this level. Nuclei were counterstained with Hoechst 33258 (blue). Scale bar: 20 µm, valid for all panels.

Control cell lines and clones were stained for Ki-67 (a marker of cells engaged in the cell cycle) under proliferation and 10 days after differentiation conditions in order to know their proliferative state. Differentiation of both control and Bcl-X_L_ hVM1 cell lines implies a reduction in the number of Ki-67^+^ cells of around 20% by day 7, being this number further reduced (80%) one week later (Figure S2 in ref. 20). Similar results were obtained for hNS1 cells [37]. In the present experiments hVM1- Bcl-X_L_ cells showed that 50% of the total cells down-regulated Ki-67 after differentiation (d10), as predicted (Figure 3C). Therefore, we have considered a reduction of about 50% of the cells expressing Ki-67 following 10 days of differentiation as an acceptable threshold. Since this is a short-term experiment (10 days) we expect down-regulation to further increase during longer differentiation times, according to ref. 8. As shown in Figure 3 B, C it is only clone #32 the one showing a near to 50% reduction, being clones # 1, 2, and 30 other examples of clones showing substantial Ki-67 downregulation (Figure 3C and Table 1).

### Nestin Expression Downregulation with Differentiation

The percentage of Nestin positive cells should parallel the data obtained for Ki-67. According to ref. [8], differentiated hVM1 cells cultures show a marked decrease in Nestin expression during a one-month differentiation (the same is true for hNS1 cells, ref. [37]). We have analyzed by ICC the expression of nestin in the selected clones under proliferation conditions and compared these data to the expression observed after 10 days of differentiation (Figure 3D–F, percentage of the reduction in Nestin^+^ cells). We have again considered as an acceptable threshold the reduction obtained for hVM1- Bcl-X_L_. Thus, clones showing a difference equal or larger to this threshold are clone numbers 2, 3, 13 and 32 (Figure 3F and Table1).

### Differentiation and TH^+^ Neuron Generation

A next step in the screening procedure was to analyze the neurogenic capacity of the clones, aiming to identify those producing an optimal number of DAn, in comparison with the polyclonal hVM1-Bcl-X_L_ line. To this end all cultures were differentiated in vitro for 10 days, and stained for a general neuronal marker (β-III-tubulin), and TH (Figure 3G). Some of the clones (# 2, 13, 32) were clearly superior to the parental line for the generation of neurons and DAn (Figure 3H). In addition, clones # 1, 15 and 30 were close to these levels (Table 1).

### In Vivo Studies: Cell Cycle Exit, Differentiation and Behavioral Amelioration

The most challenging criterion to validate the use of any clone is its *in vivo* performance. In particular, even at the short survival time used in the present study, whether the cells stop dividing and differentiate into the required cell type, DAn in the present case. To assess this we evaluated the transplants of the clones in hemiparkinsonian rats at 2 months survival time, in comparison to the polyclonal hVM1- Bcl-X_L_ cells. Brain sections of each clone and the polyclonal cell line were stained for the following markers: a) double staining with Ki-67 and human nuclei (HuNu) to asses the proliferation of cells in the transplant; b) hNSE to study whether cells start maturing in the graft and c) Tyrosine Hydroxylase (TH), to identify DAn in the transplant.

**Figure 4:**
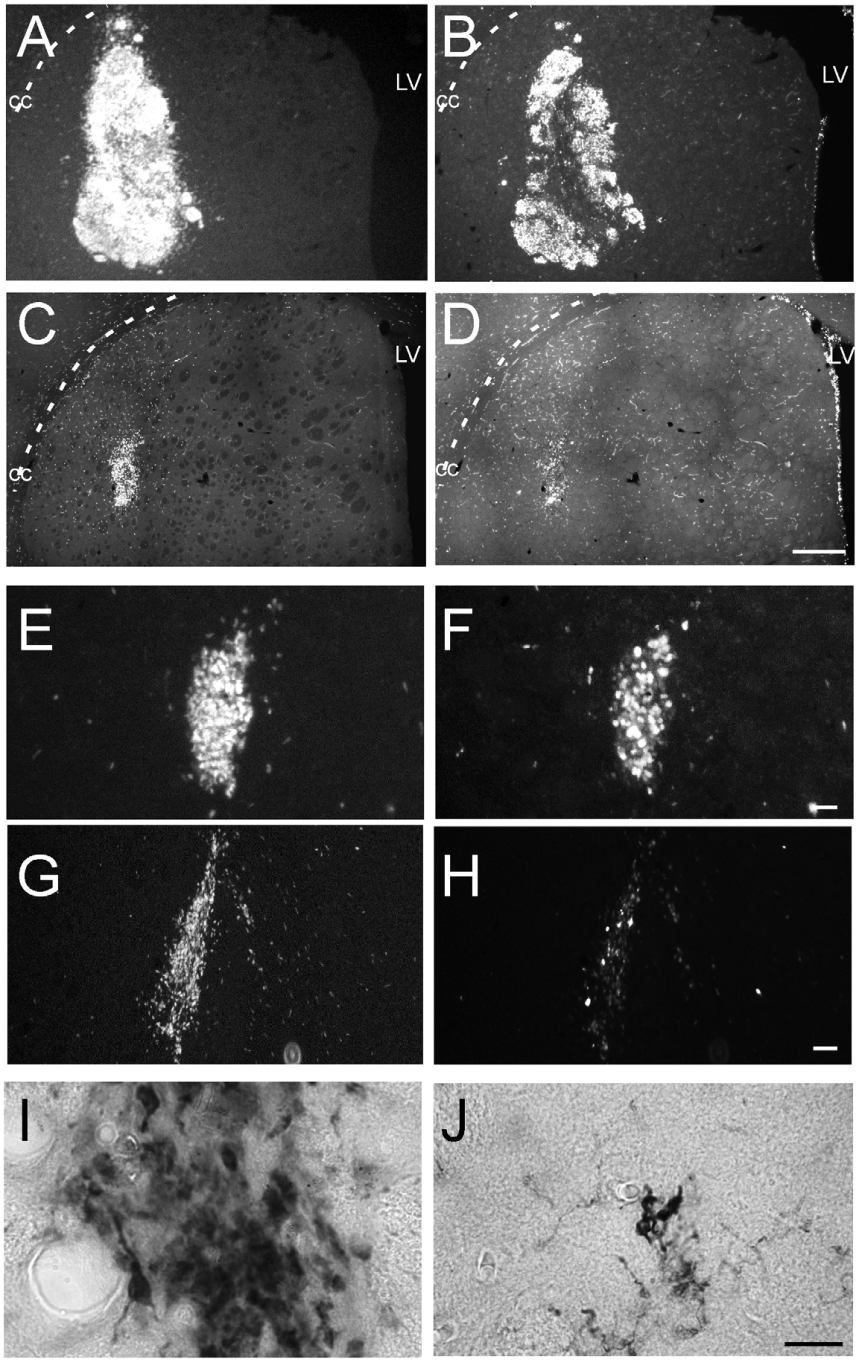
Cessation of cell division *in vivo*. A–H) Epifluorescence images of HuNu and Ki-67 immunofluorescences in sections of transplanted animals, two months post-grafting. **A–D)** Low magnification images comparing the transplants of polyclonal hVM1-Bcl-X_L_ cells (A, B) and a representative clone (# 30; C, D). HuNu allows for the identification of all human cells in the rat section and Ki-67 labels cells engaged in the cell cycle. Note the difference in graft volume between the polyclonal cells and the clone, and that the transplant of hVM1-Bcl-X_L_ cells looks heavily stained for Ki-67. Scale bar in D, 1 mm, valid for A-D. CC, corpus callosum; LV, lateral ventricle. **E–H)** Higher magnification view of two representative clones, showing poor (clone # 28; E, F) and good (clone # 32; G, H) downregulation of Ki-67. Scale bar: 40 µm, valid for E-H. **I–J)** Details of neurons differentiated in the transplants of clone 32, immunostained for hNSE (I) or TH (J). Scale bar in J: 20 µm, valid for I, J.

Anatomy of the different grafts showed, in general, a rather compact mass of cells located in the striatum around the injection site (Figure 4). This is probably due to the short post-grafting time and limited differentiation. Please note that the polyclonal hVM1-Bcl-X_L_ cells generate a less integrated graft, heavily stained for Ki-67 (Figure 4A–D, see also Figure 1). Clones were semi-quantitatively scored for the reduction of proliferating cells, as indicated in Table 1. Examples of clones showing a majority of the cells as Ki-67^+^ (score 1) and others with an evident reduction of Ki-67^+^ cells in comparison to HuNu^+^ cells (score 3) are shown in Figure 4 E–H. Even when the clones were studied at a short post-grafting time, in some of them (Table 1) a positive staining for a mature neuronal marker such as hNSE and also TH^+^ neurons were easily detected (Figure 4 I, H).

**Figure 5:**
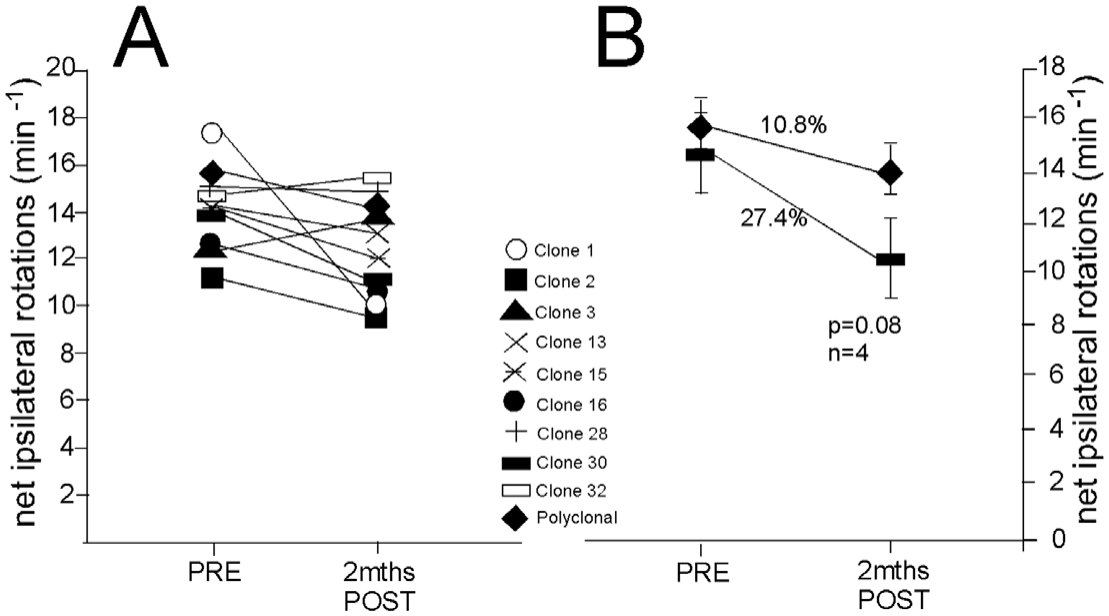
Assessment of drug-induced rotation. A) Amphetamine-induced net ipsilateral rotations before (PRE) and 2 months after transplantation (POST). Note the trend for a partial recovery observed in the majority of animal groups. Since the present work required examination of many clones, the number of animals per group was kept to a minimum (3–5) and thus non-significant trends are observed. **B)** Clone 30 transplants were twice as effective than the polyclonal hVM1-Bcl-X_L_ cel grafts in inducing rotation compensation.

*Amphetamine* induced rotation tests were additionally carried out in all groups of animals at 7 weeks post-lesion. Following *amphetamine* administration, all the grafted groups showed net ipsilateral rotation for the duration of the experiment to a varying extent (Figure 5A). Grafts of hVM1-Bcl-X_L_ cells reduced rotation as previously described [19], [20] and many of the clones also showed the same trend. Note that one of the best clones, even in the absence of increased Bcl-X_L_ levels, did reduce rotation by 27%, close to what is expected for a functional graft. Furthermore, this clone #30 is clearly superior to its parental cell line (control hVM1 cells, refs. [19], [20]).

**Figure 6:**
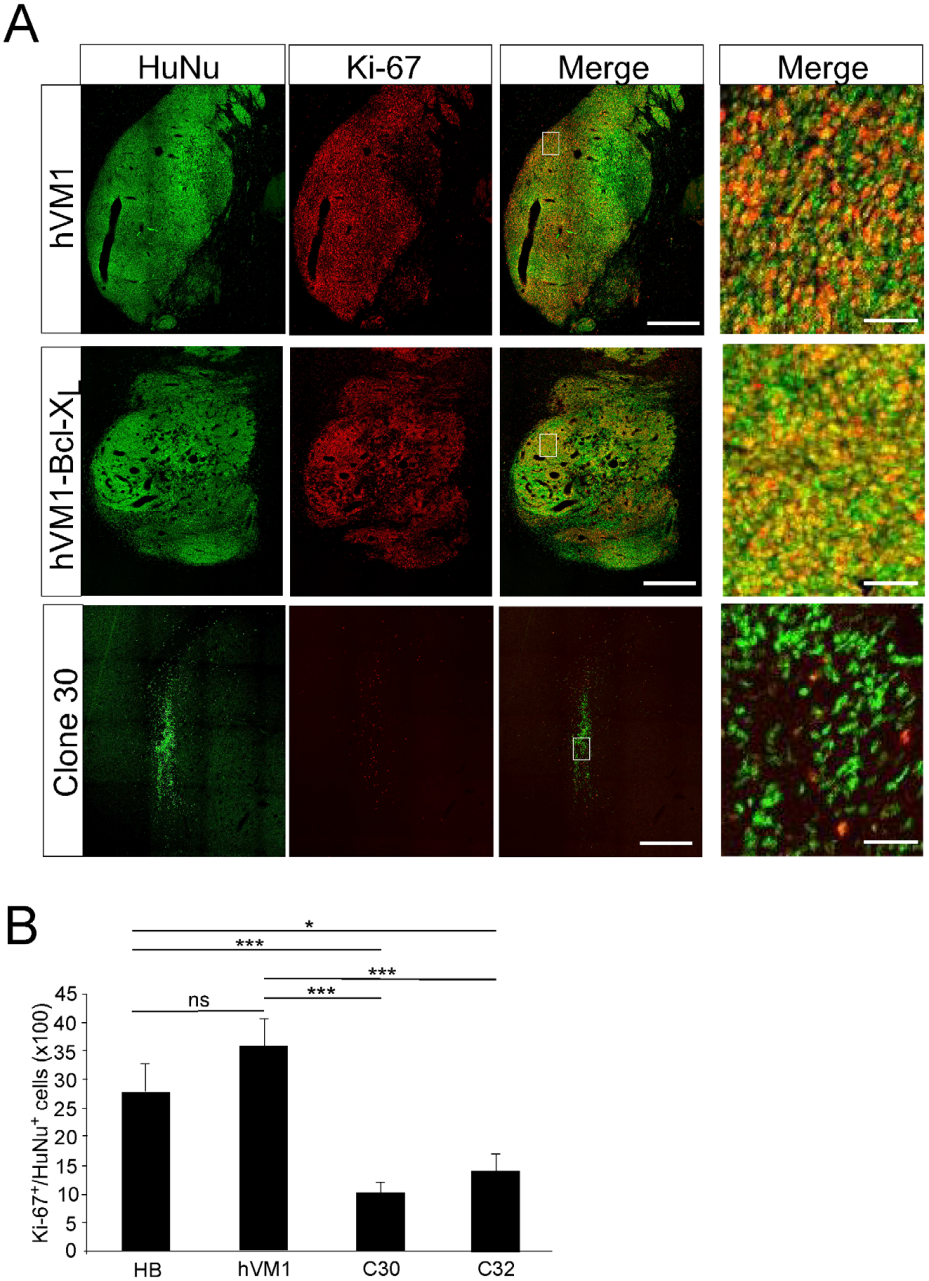
Quantitative analysis of cell cycle exit in transplanted hVM1 cells. The photomicrographs are z-stack projections of sections stained for HuNu and Ki-67 through transplants of control hVM1 and hVM1-Bcl-X_L_ cells, compared to one of the selected clones (# 30). The images in the right-hand column are high magnifications of the boxed areas to better appreciate individual nuclear staining. Scale bar corresponds to 500 µm (left three columns of images), and 20 µm (right column, high-power views). The histogram at the bottom shows the percentage of human cells (HuNu^+^) engaged in cell cycle (Ki-67^+^). Note that Bcl-X_L_ does not change the percentage of proliferating cells, and that both clones are different form the polyclonal lines. Data are expressed as the mean±SEM; *** p<0.001; * p<0.05; ns p>0.05, Student t-Test.

Summing up, and according to the majority of *in vitro* and *in vivo* criteria analyzed so far, clones # 30 and 32 are the ones showing optimal features (Table 1). Therefore, we focused on these two clones and quantified under the confocal microscope the degree of cell cycle exit at 2 months post grafting, in comparison to the two polyclonal hVM1 cell lines, either control or the Bcl-X_L_ overexpressing one. The control naïve cell line was included in this quantification since it is the perfect matching control for the clones. However, we were also interested in knowing if Bcl-X_L_ could influence the rate of exit from the cell cycle. Of note, in an *in vitro* recent study [8] we have concluded that Bcl-X_L_ does not affect the maturation rate of DAn generated from hVM1 NSCs. In another *in vivo* study and based on RT-Q PCR data [20], it seemed that maturation was more complete in Bcl-X_L_ cells at 2 months post grafting when compared to the naïve control hVM1 cells. In the present study, using quantitative histolgy, at the sampling frequency used, a total number of 332.407, 258.466, 30.238, and 13.061 HuNu^+^ cells were identified (and analyzed for Ki-67 co-expression) in Bcl-X_L_ overexpressing and non overexpressing hVM1 cell grafts and in clones # 30 and 32 cell grafts, respectively. Considering that in all animal groups the same number of cells were transplanted (3×10^5^), the one order of magnitude in difference in the total cells found in the brains may be the result of continued cell division of the polyclonal lines. A simple survival effect of Bcl-X_L_ does not account for the large difference found in total cell numbers. In accordance with the previous *in vitro* studies [8], Bcl-X_L_ does not affect the cell cycle exit *in vivo* (Figure 6, comparison between the two polyclonal lines). In the case of the polyclonal cell lines, a 25–35% of the HuNu^+^ cells were engaged in cell cycle (Ki-67^+^). The clones analyzed here showed a better rate of cell cycle exit, around 10–15% (co-localization of Ki-67 and HuNu, 2 months post-grafting; hVM-BclX_L_ = 27.78±5.04%, hVM1 = 35.79±4.83%; clone #30 = 10.28±1.79%; clone #32 = 14.17±2.88%) (Figure 6B).

### Concluding Remarks and Future Directions

Previous work and present results regarding v-*myc* immortalized cells show a down regulation of v-*myc* levels upon differentiation both *in vitro* and *in vivo*, along with cell integration into the host tissue following transplantation. Downregulation of *v-myc* is essential for the cells to differentiate normally, thus allowing neuron generation and maturation, and, in the context of regenerative medicine, to explore the potential of neuron replacement in neurodegeneration models, such as those for PD. Even when the use of immortalized cells (and therefore the selected clones # 30 and 32 identified here) will be limited to *in vitro* and pre-clinical *in vivo* research, the availability of cell lines with optimal properties will enable researchers to understand a major unresolved issue: whether human NSCs of VM origin are capable of replacing DAn in the denervated striatum in the long-term and can exert functional effects, namely, behavioral amelioration. If that would be the case, it is guaranteed that similar cells to the ones studied so far will be developed for clinical applications. For that use, the cells should be re-derived and characterized under clinically acceptable conditions, and by that time, the use of more sophisticated genetic engineering tools is envisaged to control integration and expression of *v-myc*, or to remove the transgene before transplantation [18].
